# Machine Learning Driven Synthesis of Cobalt Oxide Entrapped Heteroatom-Doped Graphitic Carbon Nitride for Enhanced Oxygen Evolution Reaction

**DOI:** 10.1371/journal.pone.0324357

**Published:** 2025-06-11

**Authors:** Abdullah Akhdhar, Abdullah S. Al-Bogami, Waleed A. El-Said, Farhan Zafar, Naeem Akhtar

**Affiliations:** 1 College of Science, Department of Chemistry, University of Jeddah, Jeddah, Saudi Arabia; 2 Institute of Chemical Sciences, Bahauddin Zakariya University (BZU) Multan, Pakistan; National Chung Cheng University, Taiwan & Australian Center for Sustainable Development Research and Innovation (ACSDRI), AUSTRALIA

## Abstract

Developing highly efficient electrocatalysts for the oxygen evolution reaction is hindered by sluggish multi-electron kinetics, poor charge transfer efficiency, and limited active site accessibility. Transition metal-based electrocatalysts, such as cobalt oxides, have shown promise. However, poor charge transfer efficiency, limited active site accessibility, and suboptimal interaction with support materials have lowered their oxygen evolution reaction performance. Additionally, optimization of materials remains a complex task, often relying on trial-and-error approaches that do not clearly understand the key features that govern oxygen evolution reaction performance. In this study, we have addressed these challenges through machine learning, which enables the systematic design and optimization of electrocatalysts. By leveraging machine learning, we have developed a highly effective cobalt oxide nanocrystal-based electrocatalyst embedded within sulfur and phosphorus-doped carbon nitride. The homogeneous distribution of cobalt oxide nanocrystals on the sulfur and phosphorus-doped carbon nitride substrate further improves the accessibility of active sites during electrochemical reactions, leading to enhanced oxygen evolution reaction performance. The cobalt oxide sulfur and phosphorus-doped carbon nitride catalyst has shown promising oxygen evolution reaction activity, characterized by a low overpotential of 262 mV, a Tafel slope of 66 mV dec ⁻ ¹, and a high electrochemically active surface area of 140.58 cm². These results highlight the synergistic interaction between cobalt oxide and sulfur and phosphorus-doped carbon nitride, which contributes to the catalyst’s superior electrocatalytic performance and provides a promising pathway for the design of advanced oxygen evolution reaction catalysts through machine learning-guided material optimization.

## 1. Introduction

Recently, environmental degradation due to the excessive use of fossil fuels to overcome the energy crisis has driven the scientific community toward the development of sustainable energy resources [1]. Among renewable energy resources, electrochemical water splitting has gained significant attention due to its ability to produce high-calorific hydrogen [2,3]. Electrocatalytic water splitting, a critical process for chemical energy conversion, involves two half-reactions: the oxygen evolution reaction (OER) and the hydrogen evolution reaction (HER) [4–6]. The OER is challenging due to its complex four-electron transfer process, converting two water molecules into one oxygen molecule [7,8]. Effective OER catalysts must enhance water adsorption, dissociation, charge transfer, and oxygen release to achieve high reaction rates and energy efficiency, necessitating low overpotentials for optimal performance [9,10].

Although noble metals such as iridium (Ir) and Ruthenium (Ru) exhibit excellent OER performance, their high cost has prohibitive widespread application [11]. Consequently, significant research has been directed toward developing cost-effective yet efficient alternatives [12–14]. In this regard, cobalt oxide (Co_3_O_4_) has emerged as a promising material due to its unique structural and electronic properties. Co_3_O_4_ with its mixed-valent spinel structure (Co II, III)) enhances electron transfer kinetics during the OER process [15,16]. Compared to other metal oxides (CuO, MnO_2_, and NiO), the spinel structure of Co_3_O_4_ provides a high density of both octahedral and tetrahedral sites, resulting in a large number of catalytic active sites that facilitate efficient reactions [17,18]. Its Co(III) ions in the octahedral sites are particularly important for activating water molecules, thus promoting efficient OER. Additionally, Co_3_O_4_ exhibits high stability under harsh electrochemical conditions, making it more reliable than other alternatives such as MnO_2_ and NiO. However, challenges such as aggregation and low conductivity can limit the efficiency of Co_3_O_4_ by restricting access to active sites and impeding electron and proton transport during the oxidation process [19].

To address these limitations, there is a need to integrate Co_3_O_4_ with conductive substrates such as carbon nitride (g-C_3_N_4_), carbon nanotubes, and graphitic carbon [20,21]. This integration not only improves the material’s conductivity but also optimizes its electronic structure, enhancing charge transfer and stability during the OER [21]. Moreover, the synergistic effect between Co_3_O_4_ and doped or supporting materials can further enhance catalytic performance. The interaction between Co_3_O_4_ and elements like sulfur, phosphorus, or nitrogen in g-C_3_N_4_ promotes the formation of Co − N bonds, which are known to enhance catalytic activity. This synergy significantly improves the electronic properties of Co_3_O_4_, making it a more efficient electrocatalyst for OER. Doping g-C_3_N_4_ with heteroatoms (e.g., I, P, S, and B) can improve its conductivity by altering its electronic structure, enhancing charge transfer, and making it a valuable substrate for catalysts in the OER [22]. Additionally, the high nitrogen content in g-C_3_N_4_ offers numerous metal ion anchoring sites, which are beneficial for catalytic applications. Co-N interactions akin to Fe-N bonds have been shown to enhance photo electrocatalytic water splitting [23]. For example, Zou et al. demonstrated a significant increase in electrocatalytic activity with the formation of Co − N bonds between g-C_3_N_4_ and Co(OH)_2_ [24,25]. Here, sulfur and phosphorus-doped g-C_3_N_4_ (SP-CN) was employed as a support framework for Co_3_O_4_ resulting in highly effective OER electrocatalysts.

Recently, machine learning (ML) has garnered significant attention in materials science and catalysis due to its advanced predictive capabilities [26]. ML helps the researchers to optimize the material properties such as adsorption energy, and active sites to design highly efficient electrocatalysts [27,28]. ML’s finely tuned variables and identifying critical factors influencing electrocatalytic activity significantly reduce the time and costs typically associated with conventional trial-and-error techniques [29,30]. Moreover, ML not only accelerates the discovery and optimization of electrocatalysts but also provides a more systematic and data-driven approach to understand the underlying mechanisms that govern electrocatalytic reactions [31]. This holistic approach is paving the way for the next generation of electrocatalysts, which are both more efficient and economically viable, contributing significantly to the advancement of sustainable energy technologies [32,33].

Inspired by the issues mentioned above, we have employed ML as a novel approach to optimize and design a robust Co_3_O_4_/SP-CN-based nanocomposite. This work introduces a novel Co_3_O_4_/SP-CN composite with S and P doping and also highlights ML as an innovative approach to finely tune the material’s properties. The integration of Co_3_O_4_ with SP-CN facilitates the formation of Co − N bonds, enhancing charge transfer rates. The designed composite (Co_3_O_4_/SP-CN) has shown high electrocatalytic activity by exhibiting low overpotential (262 mV), and a Tafel slope of 66 mV dec ⁻ ¹ that could be ascribed to the sulfur and phosphorus-doped, defect-rich g-CN structure include its strong interaction with the electrode and a high density of catalytic active sites. The uniform distribution of Co_3_O_4_ nanocrystals on SP-CN resulted in a highly conductive catalyst with minimal Co_3_O_4_ agglomeration. Consequently, the OER activity of the Co_3_O_4_/SP-CN composites was significantly enhanced. Additionally, the even dispersion of Co_3_O_4_ nanocrystals on SP-CN increased the availability of active sites for OER, thereby boosting overall catalytic performance.

## 2. Experimental section

### 2.1 Chemicals and reagents

All the chemicals and reagents, i.e., ethylene glycol (99.9%), sodium nitrate (${NaNO}_{3}$, 99.9%), thiourea (99.9%), cobalt(II) nitrate hexahydrate (Co(NO_3_)_2_.6H_2_O, 99.9%), diammonium hydrogen phosphate (NH_4_)_2_HPO_4_, 99%), nitric acid (${HNO}_{3}$, 98%), and ammonium hydroxide (NH_4_OH, 25–28%) were purchased from Sinopharm Chemical Reagent Co. (Shanghai, China).

### 2.2 Synthesis of g-CN and SP-CN

SP-CN was fabricated via a reported approach [34]. In this process, 20 mg of (NH_4_)_2_HPO_4_ was utilized as the phosphorus source, combined with 5.0 g of thiourea in an alumina crucible. The mixture was then subjected to thermal treatment in the air atmosphere, heating to 550°C at a rate of 10°C/min and maintaining this temperature for one hour; this step induced ammonia release through thermal polycondensation [35,36]. After the heating phase, the crucible was allowed to cool to room temperature inside the oven. The obtained product underwent three rinses with distilled deionized water and 100% ethanol, followed by air-drying at 50°C for 24 hours. Subsequently, the dried material was finely ground using a pestle and mortar.

### 2.3 Synthesis of Co_3_O_4_ nanoparticles (Co_3_O_4_ NPs)

Cobalt oxide nanoparticles (Co_3_O_4_ NPs) were synthesized via a conventional hydrothermal method [37,38]. First, 1.5 g of cobalt chloride hexahydrate (CoCl_2_·6H_2_O) was dissolved in 30 mL of deionized water. The CoCl_2_ solution was mixed with the ammonium hydroxide solution while stirring continuously at 250–350 rpm. This mixture was transferred to an autoclave and subjected to hydrothermal treatment at 160°C for 8 hours. Following the reaction, the autoclave was allowed to cool to 25°C. The resulting product was a dark powder comprising Co_3_O_4_ NPs.

### 2.4 Synthesis of cobalt oxide (Co_3_O_4_) entrapped in SP-CN (Co_3_O_4_/SP-CN)

Co_3_O_4_/SP-CN was synthesized according to the previously reported approach [34]. Briefly, 50 mg of SP-CN and 50 mg of Co_3_O_4_ NPs were dissolved in ethanol. The solution was then subjected to continuous magnetic stirring and heating until the solvent was fully evaporated. After drying at 50°C for 24 hours, the remaining residue was subjected to heat treatment at 550°C for three hours. The resulting product was a brown composite of Co_3_O_4_/SP-CN.

## 3. Results and discussion

**Fig 1:**
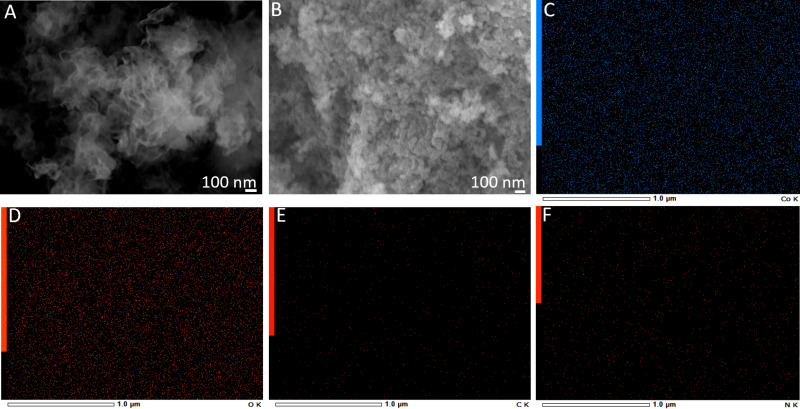
SEM images of (A) SP-CN, (B) Co3O4/SP-CN. EDX mapping of (C) Co, (D) O, (E) C, and (F) N.

The surface morphology of SP-CN and Co_3_O_4_/SP-CN composite was examined using scanning electron microscopy (SEM) (Fig 1). SEM images of SP-CN showed the presence of two-dimensional (2D) sheets with an irregular, flat structure and numerous wrinkles (Fig 1A). In contrast, SEM images of Co_3_O_4_/SP-CN (Fig 1B) revealed well-dispersed Co_3_O_4_ nanoparticles (NPs) across the 2D sheets, with sizes ranging from 20 to 40 nm in diameter. The size of Co_3_O_4_ nanocrystals plays a significant role in the OER, as smaller NPs with their high surface-to-volume ratio tend to have higher surface area, enhancing their catalytic activity in electrochemical reactions. Additionally, SEM images highlight the homogeneous dispersion of Co_3_O_4_ NPs on SP-CN sheets. This homogeneous dispersion will ensure optimal exposure of active sites leading to efficient electron transfer kinetics. Additionally, EDX mapping (Fig 1C-1F) confirms the successful fabrication of the Co_3_O_4_/SP-CN composite. The EDX mappings for Co (Fig 1C), O (Fig 1D), C (Fig 1E), and N (Fig 1F) indicate a homogeneous distribution of these elements across the surface.

**Fig 2:**
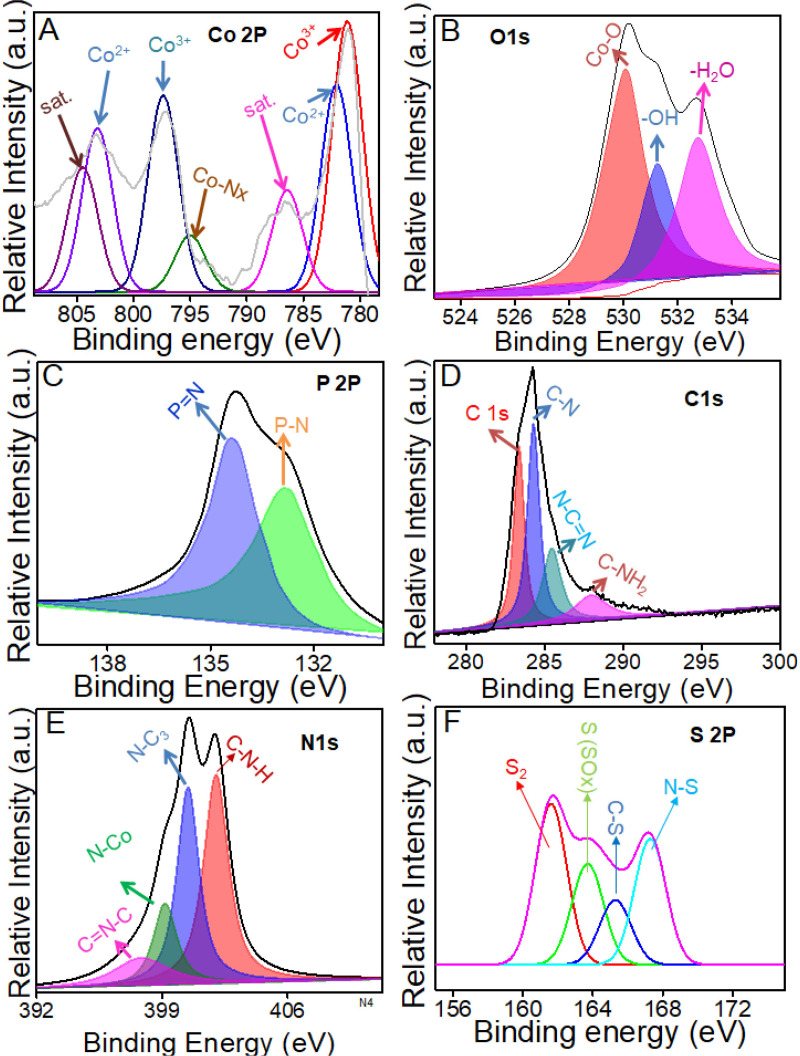
Representing XPS spectra of Co3O4/SP-CN (A) Co, (B) O, (C) P, (D) C, (E) N, and (F) S.

The surface elemental composition and chemical states of the Co_3_O_4_/SP-CN composite were analyzed using X-ray photoelectron spectroscopy (XPS). The XPS spectra showed the presence of P, S, C, N, O and Co without any other contaminants (Fig 2). The Co 2p spectra displayed peaks for Co^3+^and Co^2+^ at 781.1 & 797.5 eV, and 782.7 & 801.3 eV, respectively, along with satellite peaks at 804.1 and 786.5 eV (Fig 2A), indicating higher binding energies. Peaks at 779.6 and 795.0 eV were assigned to the Co-Nx structure in Co_3_O_4_/SP-CN (Fig 2A). The O 1s spectra showed peaks at 530.1, 531.3, and 532.1 eV, corresponding to surface H_2_O, adsorbed oxygen, and lattice oxygen in Co_3_O_4_, respectively (Fig 2B). The P 2p spectra indicated binding energy maxima for P-O, P = N, and P-N bonds at 133.2, 134, and 135 eV, respectively. While higher binding energies for P = N, and P-N bonds compared to P-C coordination suggested phosphorus substitution for carbon in the triazine rings [39,40] (Fig 2C). Deconvolution of the C 1s XPS spectra for Co_3_O_4_/SP-CN identified peaks at 284.6, 286.3, 288.1, and 289.0 eV, corresponding to C-N, and Sp^2^-bonded C in N-C = N, respectively [41] (Fig 2D). The N 1s XPS spectra for SP-CN displayed peaks at 397.8, 400.4, and 402.01 eV for C = N-C, N-C_3_, and C-NH_2_, respectively, with an additional peak at 399.1 eV indicating the Co-N bond in Co_3_O_4_/SP-CN (Fig 2E). Fig 2F confirms the presence of S with different binding interactions. These findings align with the literature, confirming the successful synthesis of the Co_3_O_4_/SP-CN composite [34].

**Fig 3:**
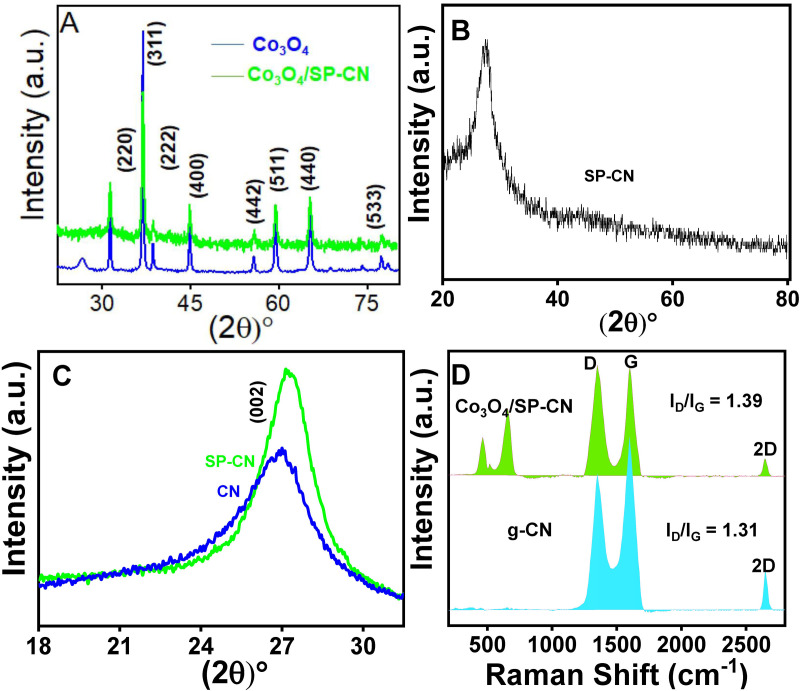
Representing XRD pattern of (A) Co3O4 and Co3O4/SP-CN, (B)SP-CN, (C) SP-CN, CN and (D) Raman spectra of g-CN, Co_3_O_4_/SP-CN.

X-ray diffraction (XRD) was used to assess the crystallinity and phase purity of Co_3_O_4_ and Co_3_O_4_/SP-CN [34] (Fig 3A). The XRD pattern confirmed the presence of Co_3_O_4_, with diffraction planes at 2θ angles of 31.37°, 36.87°, 38.67°, 44.87°, 55.62°, 59.56°, 65.47°, and 77.41° corresponding to the (220), (311), (222), (400), (442), (511), (440), and (533) planes, respectively, in agreement with JCPDS# 74–2120 [34]. The presence of SP-CN was confirmed by peaks at 2θ of 13.0° and 27.7°, corresponding to the (002) diffraction plane (Fig 3B). Sulfur and phosphorus in CN caused a positive shift in the 2θ value, attributed to increased interplanar distance and lattice distortions (Fig 3C) [42,43].

Raman spectroscopy was employed to analyse the graphitic carbon content in Co_3_O_4_/SP-CN and SP-CN. The Raman spectra (Fig 3D) showed two prominent bands: the G-band at approximately 1590 cm ⁻ ¹, indicative of quasi-graphitic carbon layers, and the D-band at around 1354 cm ⁻ ¹, indicative of disordered carbon. The D-band represents structural disruptions, while the G-band corresponds to ${Sp}^{2}\;$-hybridized graphitic layers [44,45]. The degree of graphitization is often quantified by the intensity ratio of the D-band to the G-band (ID/IG). Co_3_O_4_/SP-CN exhibited a higher ID/IG ratio of 1.39 compared to SP-CN, indicating increased graphitization and surface defects. The presence of a 2D band suggested that co-doping with sulfur and phosphorus promoted ${Sp}^{3}\;$hybridization in some graphitic layers, enhancing conductivity and electron transport [46,47].

The Fourier transform infrared (FTIR) spectroscopy was carried out to study the stretching and bending vibrational modes of Co_3_O_4_ and Co_3_O_4_/SP-CN (S1 Fig). FTIR spectrum of Co_3_O_4_ has shown two prominent bands at 571 cm ⁻ ¹ and 664 cm ⁻ ¹, which are attributed to the stretching vibrations of Co³⁺ and oxygen, confirming the presence of spinel Co_3_O_4_. In the case of Co_3_O_4_/SP-CN, a band at 1630 cm ⁻ ¹ corresponding to C = N stretching, along with absorption peaks in the 1250–1400 cm ⁻ ¹ range, indicate C–N stretching typical of tertiary amines. These features suggest the presence of a conjugated C–N framework within graphitic carbon nitride (g-C_3_N_4_), confirming the formation of an extended π-conjugated structure. Furthermore, peaks in the 1280–1350 cm ⁻ ¹ are attributed to P = N bonds, while those between 1050–1150 cm ⁻ ¹ correspond to C–S or S–N bonds, confirming the successful integration of S and P into the material’s structure.

The BET surface analysis of Co_3_O_4_/SP-CN and SP-CN was carried out using nitrogen (N_2_) adsorption-desorption isotherms to evaluate its surface area and pore volume (S2 Fig). The N_2_ adsorption-desorption isotherm displayed a typical IV-type isotherm with a distinct hysteresis loop, indicating mesoporous characteristics. The Co_3_O_4_/SP-CN has shown a high specific surface area of 141 m²/g compared to SP-CN (81 m²/g). This high surface area of Co_3_O_4_/SP-CN with porosity will facilitate better electron and ion transport leading to fast OER reaction kinetics.

### 3.1. Optimization of material composition to minimize the overpotential through ML

**Fig 4:**
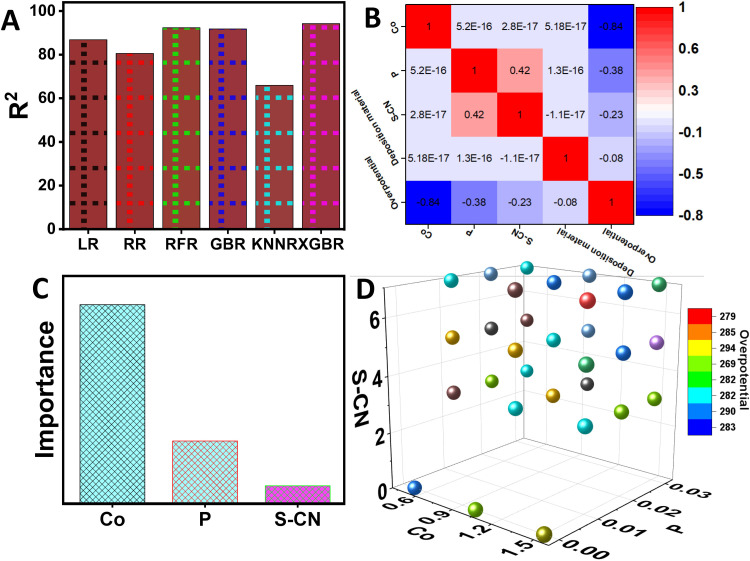
Comparative analysis of electrocatalyst performance. (A) Bar chart showing key experimental variables affecting OER activity. (B) Correlation matrix heatmap with color-coded strengths. (C) importance feature graph, and (D) 3D scatter plot linking SP-CN doping levels to OER efficiency, indicating variable influence.

Several ML regression models were employed to design highly efficient electrocatalysts with minimized overpotential. These models included linear regression (LR), K-nearest neighbors regression (KNNR), random forest regression (RFR), ridge regression (RR), gradient boosting regression (GBR), and extreme gradient boosting regression (XGBR). The ML models were trained on the experimental dataset to optimize the concentration of Co, P, S-CN, and the amount of material deposited, aiming to predict overpotential. Each ML model was trained on preliminary experimental dataset to effectively minimize overpotential as a function of the variables mentioned. The predictive capabilities of each ML model are illustrated in Fig 4A. Among these models, the XGBR model stands out, showing the most effective optimization and prediction of low overpotential, with a high coefficient of regression (R² = 94.25%). This model’s strength lies in its ability to handle complex, nonlinear relationships and interactions between experimental variables, without assuming linearity, making it well-suited for modeling intricate experimental data and accurately predicting overpotential in electrocatalyst design. The RFR and GBR models also demonstrated strong predictive performance, with R² values of 92.31% and 91.79%, respectively, highlighting their robustness in capturing nonlinear relationships. In contrast, models like LR, RR, and KNNR exhibited higher Root Mean Square Errors (RMSEs) and lower R² values, suggesting potential overfitting and reduced predictive accuracy (S1 Table). These findings emphasize the XGBR model’s capability to generalize effectively across various scenarios, making it a powerful tool for optimizing material composition and enhancing electrocatalyst efficiency (S3 Fig).

Moreover, the Pearson correlation matrix provides further insight into the relationships between Co, P, S-CN, deposited material, and overpotential, which is crucial for designing efficient electrocatalysts (Fig 4B). The analysis reveals a strong negative correlation between Co and overpotential (−0.84), indicating that higher concentrations of Co significantly reduce overpotential. P shows a moderate negative correlation with overpotential (−0.38), suggesting a decrease in overpotential with increasing P concentration. Meanwhile, S-CN and the material deposited exhibit weak negative correlations with overpotential (−0.23 and −0.08, respectively), indicating their minimal impact on overpotential. These results highlight the significant role of Co in lowering overpotential, while P and S-CN have relatively minor effects.

Furthermore, feature importance analysis identifies Co as the most critical factor in controlling overpotential, followed by P, with S-CN having the least influence (Fig 4C). A 3D scatter plot has been utilized to determine the optimal combination of Co, P, S-CN, and deposited material that minimizes overpotential, thereby enhancing the catalytic efficiency of the synthesized materials (Fig 4D). This data-driven approach refines the synthesis process by providing precise control over material properties, significantly advancing catalyst performance in OER applications. The method not only simplifies the optimization process but also establishes a solid foundation for the rational design of high-performance catalytic material. The 3D scatter plot specifically identifies the optimal concentrations of Co (1.5), P (0.02), S-CN (5), and deposited material (1.5), corresponding to a minimum overpotential of 262 mV. This ML-driven analysis underscores the importance of optimizing Co, P, S-CN and depositing material concentrations for OER applications.

### 3.2. Electrocatalytic activity of Co_3_O_4_ and Co_3_O_4_/SP-CN

**Fig 5:**
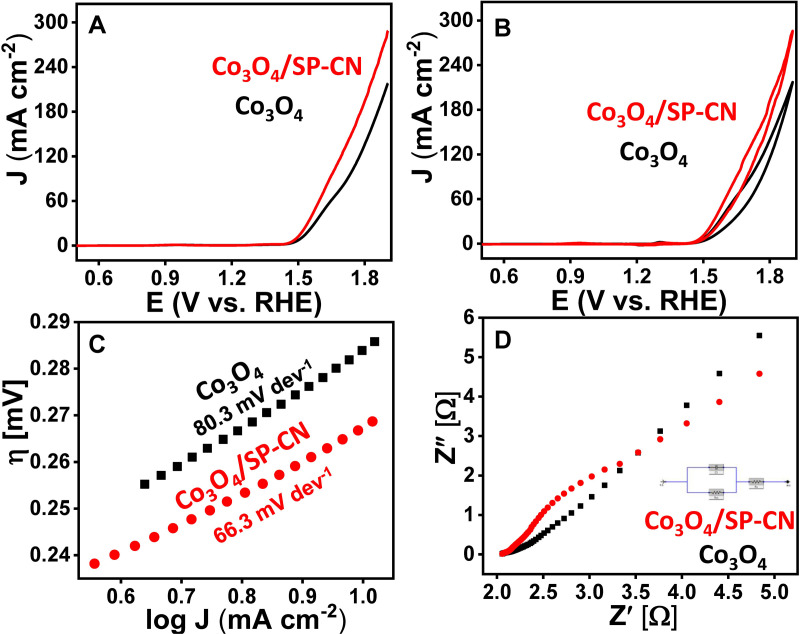
Electrocatalysis measurements of Co_3_O_4_ and Co_3_O_4_/SP-CN (A) LSV, (B) CV, (C) tafel plot, and (D) EIS.

The electrocatalytic performance of the synthesized materials was assessed using a three-electrode setup, comprising a Pt wire as the counter electrode, an Ag/AgCl electrode as the reference, and a customized nickel foam electrode as the working electrode. Linear sweep voltammetry (LSV) and cyclic voltammetry (CV) were performed at a scan rate of 100 mV/s in 1 M KOH, covering a potential range from 0 to 2 V vs. RHE, to determine the onset potential for each material (Fig 5A, 5B). The LSV results revealed that Co_3_O_4_/SP-CN exhibited a lower onset potential of 1.43 V vs. RHE compared to Co_3_O_4_, which had an onset potential of 1.52 V vs. RHE. Moreover, at a current density of 10 mA/cm², Co_3_O_4_/SP-CN demonstrated a reduced overpotential of 262 mV vs. RHE, in contrast to 303 mV vs. RHE for Co_3_O_4_. The occurrence of swirling at the electrode surface during LSV further indicated the activation. For a deeper understanding of the reaction kinetics, the Tafel slope was derived by plotting the logarithm of current density (log j) against overpotential (Fig 5C). The data indicated that Co_3_O_4_/SP-CN had a lower Tafel slope of 66 mV/dec compared to Co_3_O_4_, which had a Tafel slope of 80.3 mV/dec. The results indicate that Co_3_O_4_/SP-CN promotes significantly faster and more efficient OER kinetics compared to other comparable electrocatalysts (S2 Table). This enhanced electrocatalytic performance can be attributed to the effective stabilization and facilitation of intermediate species at the uniformly distributed Co_3_O_4_ active sites.

Electrochemical impedance spectroscopy (EIS) was carried out to investigate the electron transfer resistance associated with the OER process. EIS data was fitted using a Randles equivalent circuit consisting of solution resistance (Rs), charge transfer resistance (Rct), and double-layer capacitance (Cdl), providing insight into electrochemical behavior. The EIS-fitted Nyquist plot (Fig 5D) of Co_3_O_4_/SP-CN had shown the lowest Rct (3.8 Ω) as compared to Co_3_O_4_ (4.4 Ω). This enhanced performance can be attributed to S and P co-doping, which introduces structural defects in g-CN. These defects not only improve electrical conductivity but also strengthen the interaction between SP-CN, Co_3_O_4_, and the electrode surface. Additionally, the defect-rich structure increases the density of exposed catalytic active sites, collectively promoting faster reaction kinetics and enhancing the overall OER activity.

**Fig 6:**
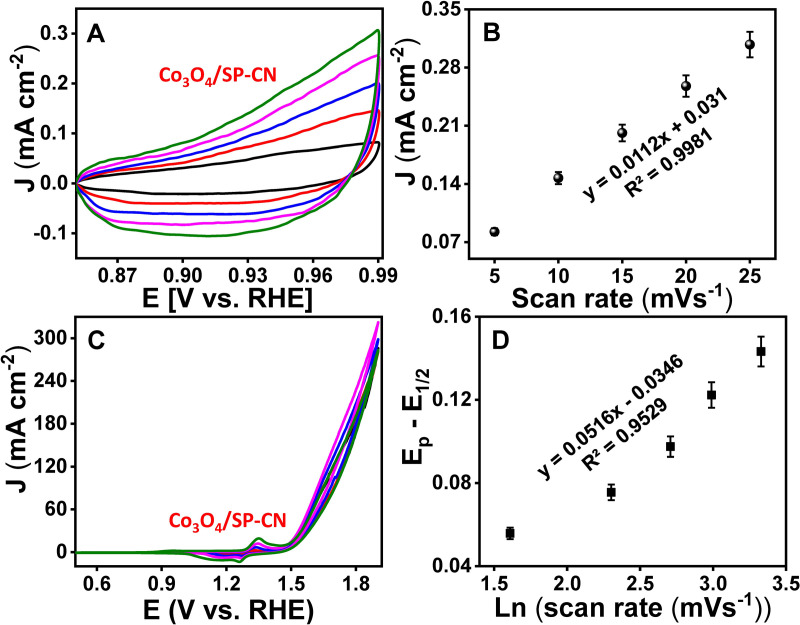
Evaluation of electrocatalytic activity (A) CV in the non-faradaic region, (B) Calibration curve Co_3_O_4_/SP-CN, (C) CV, and (D) calibration curve.

The electrocatalytic active surface area of the electrode was determined by evaluating the double-layer capacitance (*Cdl*) within the non-faradaic region through the CV performed in 0.1 M PBS at varying scan rates from 5 mV/s to 25 mV/s (Fig 6A). A linear correlation was observed between the current density and the scan rate (Fig 6B). The electroactive surface area and Cdl, calculated from the slope in Fig 6A, were 140 cm² and 5.6 mF/cm², respectively. These results indicate that SP-CN enhances the electrocatalytic performance of Co_3_O_4_ by increasing the number of accessible catalytic sites and facilitating rapid charge transfer. The improved activity is attributed to the strong Co-N binding interactions between Co_3_O_4_ and g-CN, as well as the surface charge defects introduced by sulfur and phosphorus doping.

### 3.3. Reaction kinetics and mechanistic investigations

The number of electrons involved in the OER was calculated by analyzing the relationship between the applied potential and the natural logarithm of the scan rate, which varied from 5 to 50 mV/s (Fig 6C). This evaluation was conducted following the Laviron equation (Eq. 1).


$$Ec=E_{1/2}-\left(\frac{RT}{\alpha nF}\right)*\ln\left(\frac{\alpha nF}{RTks}\right)-\left(\frac{RT}{\alpha nF}\right)*\ln(v)\;\;\;\;\;\;\;\;\;\;\;\;\;\;\;(1)$$

Slope=2.303RT/αnF (2)

In this analysis, Ec represents the reduction potential, while E_1/2_ denotes the formal potential of the metal redox process. R, T, F, and ks correspond to the gas constant, absolute temperature, Faraday constant, and average rate constant for the redox reaction, respectively. The electron transfer coefficient and the number of electrons transferred are denoted by α and n, respectively. Using Eq. 2, the electron transfer and the number of electron-proton transfers were calculated to be 3.89 and 0.812, respectively (Fig 6D). To investigate the reaction kinetics, the Laviron equation (Eq. 1) was applied to determine the ks values, providing insights into the adsorption properties of the newly developed electrodes.

CV experiments were performed for Co_3_O_4_/SP-CN and Co_3_O_4_ in 1 M KOH at scan rates ranging from 5 to 50 mV/s. The data revealed that both Co_3_O_4_/SP-CN and Co₃O₄ exhibited stable redox currents, with a linear correlation between the applied potential and the natural logarithm of the scan rate. Notably, the *ks* value for Co_3_O_4_/SP-CN (0.73 s^−1^) was higher than that of Co_3_O_4_ (0.44 s^−1^), suggesting more efficient metal binding to ^−^OH species on the electrode surface. These findings align with the proposed OER mechanism, which is supported by literature and involves a four-electron-proton transfer process accompanied by oxo-intermediate formation (Eqn. 3–6) on the electrode surface.


$${\mathrm{Co}}^{+2}2^{-}\mathrm{OH}\to \mathrm{CO}{\left(\mathrm{OH}\right)}_{2}$$ (3)

$$\mathrm{CO}{\left(\mathrm{OH}\right)}_{2}+{\mathrm{OH}}^{-}\to \mathrm{Co}-\mathrm{OOH}+H_{2}O+e^{-}$$ (4)

$$\mathrm{Co}-\mathrm{OOH}+{\mathrm{OH}}^{-}\to \mathrm{Co}-{\mathrm{OO}}^{-}+H_{2}O$$ (5)

$$Co-OO^{-}\to Co+O_{2}+e^{-}$$ (6)

Briefly, Co undergoes the oxidation from Co²⁺ to Co³⁺ on exposure to alkaline media (KOH), resulting in the formation of OER different intermediates (Co(OH)_2,_ Co-OOH, Co-OO^-^). These intermediates finally result in the evolution of O_2_ at the electrode surface [48]. This mechanism aligns with existing literature [49]. The efficient and fast production of O_2_ is supported to high surface-to-volume ratio and homogeneous dispersion of Co_3_O_4_ NPs on SP-CN sheets. This homogeneous dispersion enhnced OH⁻ adsorption due to strong interactions between the metal and the OH⁻ intermediate leading to efficient electron transfer kinetics.

**Fig 7:**
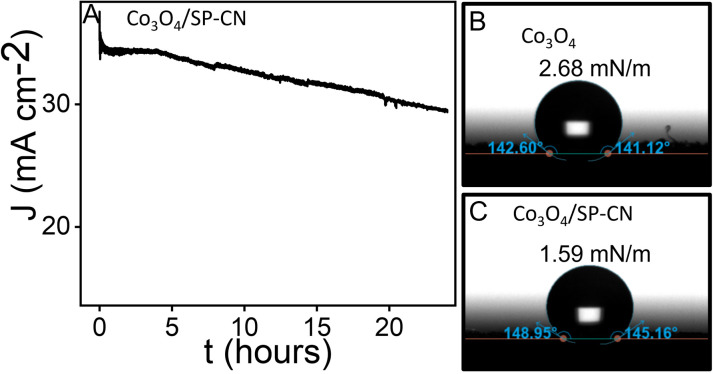
(A) Chronoamperometric measurement representing stability, (B) Contact angle measurement Co_3_O_4_, and (C) Co_3_O_4_/SP-CN.

Chronoamperometric tests conducted at 1.40 V vs. RHE over 24 hours in a 1 M KOH solution showed that Co_3_O_4_/SP-CN sustained a current density above 35 mA/cm² with minimal performance degradation (Fig 7A). However, after the stability test, the XRD pattern revealed a slight decline in peak intensity and broadening of the diffraction peaks (red), suggesting a partial loss of crystallinity (Fig S4). This change is indicative of phase instability, reflecting the material’s degradation over time. Furthermore, XPS has been carried out to investigate the structural integrity of Co_3_O_4_/SP-CN following the stability test. XPS spectra has shown minimal variations in binding energy and peak intensities, indicating that each element chemical state almost remains the same (Fig S5). Contact angle measurements of Co_3_O_4_ (Fig 7B) indicate a higher binding energy as compared to Co_3_O_4_/SP-CN (Fig 7C). The lower binding energy observed in Co_3_O_4_/SP-CN contributes to improved stability of the system. This reduction in binding energy is attributed to the strong cohesive interactions within the Co_3_O_4_/SP-CN nanocomposite, as supported by prior studies [26]. Furthermore, the OER performance of this electrocatalyst, particularly in terms of onset potential, is comparable to or exceeds that of various chalcogenides and MOF-based metal electrocatalysts reported in the literature.

## 4. Conclusions

In this study, we synthesized ML-optimized novel and cost-effective Co_3_O_4_/SP-CN-based nanocomposite by pyrolyzing thiourea and subsequently combining it with cobalt salt and diammonium phosphate. The designed composite (Co_3_O_4_/SP-CN) has shown high electrocatalytic activity by exhibiting low overpotential (262 mV), and a Tafel slope of 66 mV dec ⁻ ¹ that could be ascribed to the sulfur and phosphorus-doped, defect-rich g-CN structure include its strong interaction with the electrode and a high density of catalytic active sites. ML has identified Co as the key element controlling OER efficacy followed by P, S-CN. Additionally, chronoamperometric measurements confirmed the high stability and durability of the catalyst, further validating its potential for long-term use in OER applications.

## Supporting information

S1 File Machine learning. (DOCX)
