# Supplementary material for: Machine Learning Driven Synthesis of Cobalt Oxide Entrapped Heteroatom-Doped Graphitic Carbon Nitride for Enhanced Oxygen Evolution Reaction
Source: PLoS One. 2025 Jun 11;20(6):e0324357. doi: 10.1371/journal.pone.0324357 (PMC12157112; doi:10.1371/journal.pone.0324357)
Supplement: S1 File — (DOCX) [file pone.0324357.s001.docx]

**Supporting Information**

**Machine Learning Driven Synthesis of Cobalt Oxide Entrapped Heteroatom-Doped Graphitic Carbon Nitride for Enhanced Oxygen Evolution Reaction**

**Characterization and Electrochemical Techniques**

The phase composition was analyzed by X-ray diffraction (XRD) measurements by using a Rigaku D/max-2550 instrument equipped with a Cu-Kα radiation source (λ=1.5418 Å). Raman spectra were obtained via Raman Microscope by RENISHAW UK with excitation laser of 514nm (laser power: 100%, grating: 1800 I/mm) and laser exposure time of 10s. X-ray photoelectron spectroscopy (XPS) was conducted using a PHI Quantera SXM (ULVAC-PH) with monochromated AlKα radiation under a pressure of 4 × 10^−8^ Pa. The surface spectrum of carbon-coated ZnO was recorded, and the peak shift was evaluated for each of the surface-capping agents. For fitting, the XPSpeak41 software was used. SEM analysis using an analytical low-vacuum SEM (JEOL) model no JSM-6490LA was carried out to characterize the mean particle size and morphology of nanoparticles. Automated vacuum mode change-over was selected for this study. Accelerating voltage of microscope range was set at 20kV for each sample.


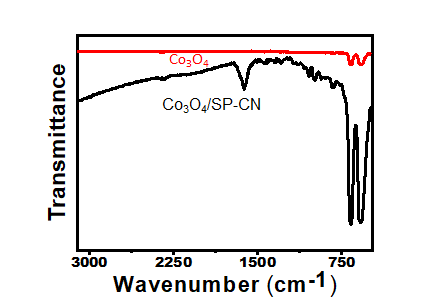


**Figure S1** shows the FTIR spectrum of Co_3_O_4_ and Co_3_O_4_/SP-CN.


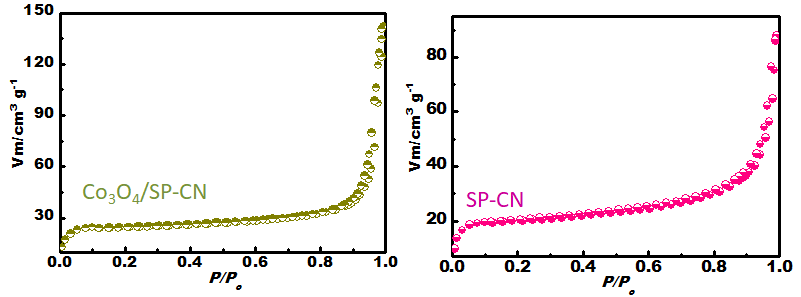


**Figure S2** shows N_2_ adsorption-desorption isotherm of (A) Co_3_O_4_/SP-CN (B) SP-CN.

All the electrochemical investigations were carried out using the Gamry interface (1010E) potentiostat. Almost all analytical electrochemical characterization was performed via three electrodes to investigate the electrochemical analysis: the working electrode, which may be bare or coated by fabricated nanoparticles, Ag/AgCl acts as a reference, and a Pt wire used as a counter electrode. Nickel foam (NF) was modified with synthesized material (Co_3_O_4_ and Co_3_O_4_/SP-CN). Co_3_O_4_/SP-CN slurry was prepared, and 5 μL was drop-casted and air-dried.

**Fabrication of the working Electrode**

Herein, working electrodes were fabricated by coating a graphitic pencil with Co_3_O_4_ and Co_3_O_4_/SP-CN. Firstly, to obtain a catalyst ink, 2 mg of as-prepared samples (Co_3_O_4_ and Co_3_O_4_/SP-CN), 100 μL of 5 wt % Nafion solution, and 200 μL of isopropyl alcohol were dispersed in 700 μL of deionized water by at least 30 min of ultrasonic processing to form a homogeneous ink. To ensure the optimal dispersion of the Co_3_O_4_ and Co_3_O_4_/SP-CN nanoparticles, ultrasonic processing was used to break up agglomerates, ensuring even distribution of the catalyst within the ink. The prepared slurry was drop-casted onto nickel foam (NF). After deposition, NF was dried at 25˚C for 8 h, giving a mass loading of 2 mg cm^-2^.

1. **Machine learning**

Machine learning was implemented using the scikit-learn package in Python 3. OER electrocatalytic activity of the designed material was predicted by employing various regression algorithms, including Linear Regression (LR), K-Nearest Neighbors Regression (KNNR), Gradient Boosting Regression (GBR), Random Forest Regression (RFR), Ridge Regression (RR), and Extreme Gradient Boosting Regression (XGBR). These algorithms were employed to predict feature-objective relationships and evaluate model performance using two statistical measures: root mean square error (RMSE) and the coefficient of regression (R²). The models were trained and tested on datasets split into 70% for training, 15% for validating and 15% for testing. The optimal OER model was determined based on the highest accuracy, characterized by the lowest RMSE and the highest R² value. Figure S2 presents the performance of various ML models in predicting overpotential, a crucial parameter in electrocatalysis and energy conversion processes. Each subplot (A-H) shows the actual overpotential on the x-axis and the predicted on the y-axis, with data points categorized into training (red squares), validation (blue circles), and test (green triangles) sets. The color-coding of data points provides a clear visualization of each model's generalization capability, with the diagonal alignment of points indicating high prediction accuracy.


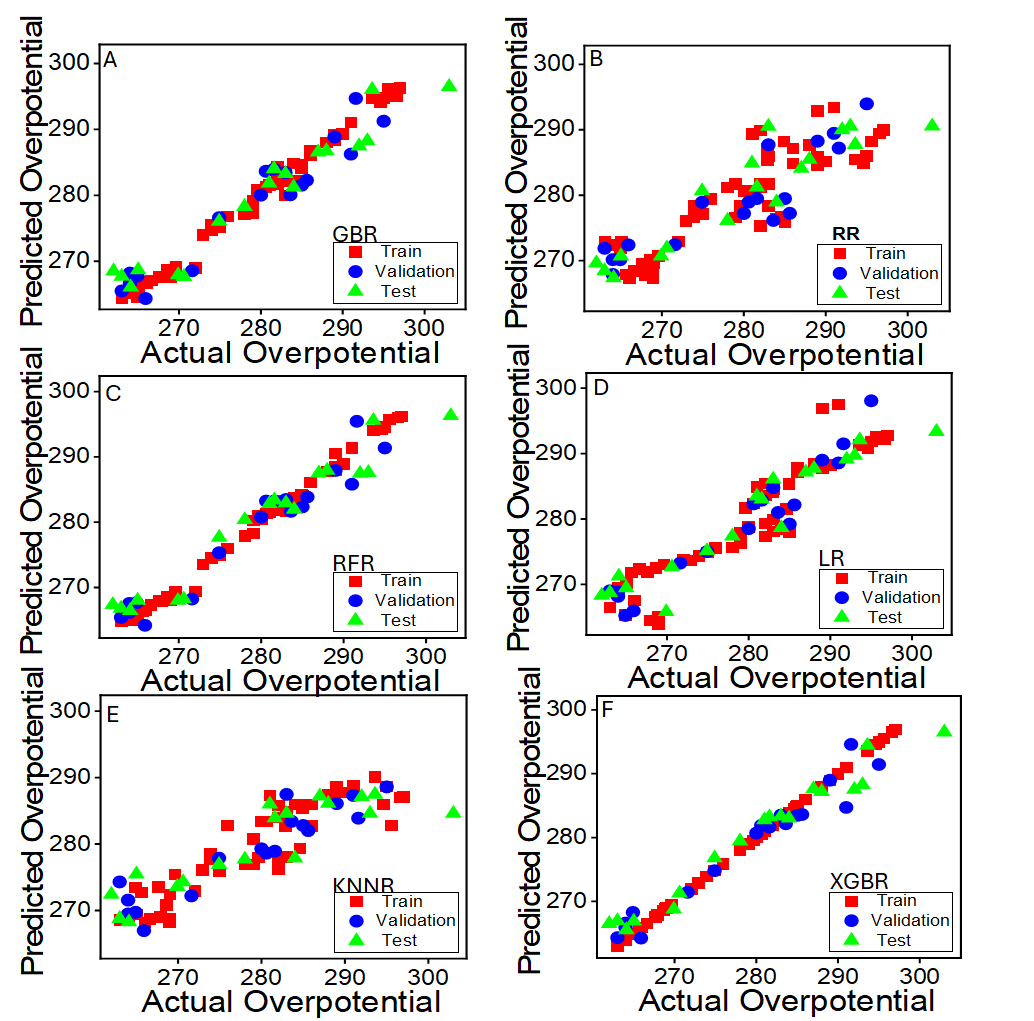


**Figure S3** represents the predictive performance of different ML models (A) Gradient Boosting Regression (GBR), (B) Ridge Regression (RR), (C) Random Forest Regression (RFR), (D) Linear Regression (LR), (E) K-Nearest Neighbors Regression (KNNR), (F) Extreme Gradient Boosting Regression (XGBR). Data points are categorized into training (red squares), validation (blue circles), and test (green triangles) sets.

1. **Electrochemical measurements**

A GAMRY electrochemical potentiostat was used for all of the measurements of electrochemical properties. Three-electrode setup was employed with a reference electrode of Ag/AgCl, working electrodes of Co_3_O_4_ and Co_3_O_4_/SP-CN, and a counter electrode of platinum wire. All of these electrodes were submerged in a 1M KOH solution, acting as an ionic electrolyte. All electrochemical studies are conducted at this workstation, including those involving cyclic voltammetry (CV), linear sweep voltammetry (LSV), electrochemical impedance spectroscopy (EIS) and chronoamperometry.

A 3:1 combination of HNO_3_ and H_2_SO_4_ was used to boil the electrochemical cell before rinsing it with ultrapure water to clean it before use. Then it was repeatedly cleaned with acetone followed by ultrapure water. Following washing, it was oven dried for 30 to 40 minutes at 80°C. After being thoroughly rinsed with ultrapure water and briefly dipped in a 20% solution of HNO_3_, the counter electrode, which was composed of Pt wire, was put into the electrochemical cell. At room temperature, all measurements were performed in a 1 M KOH electrolyte solution of pH 13.5. All the potentials were reported relative to the reversible hydrogen electrode (RHE) using the equation.

E_RHE =_ E_Ag/AgCl_ + 0.059 pH +E^˚^_Ag/AgCl_ eqn. S1

where E_Ag/AgCl_ is the potential measured against the Ag/AgCl electrode, E^˚^_Ag/AgCl_ is the standard thermodynamic potential (0.197 V) of Ag/AgCl, and E_RHE_ is the calculated potential vs. RHE.

- 1. **Electrochemical impedance spectroscopy**

To determine the charge transfer resistance (R_ct_) in the frequency range of 100 kHz to 0.1 Hz with an amplitude of 10 mV, electrochemical impedance spectroscopy (EIS) was used. The diameter of a semi-circle in the Nyquist plot illustrates R_ct_ at the electrode/electrolyte interface.

- 1. **Tafel slope calculations from the polarization curve**

Tafel graph is used to compute the catalytic activity and kinetics by considering the linear portion of the steady-state polarization curve as expressed in equation.

$ŋ=a+\left( 2.3.3\frac{RT}{ꭤnF} \right)*\log j$ eqn. S2

Here, over potential is represented by ŋ, charge transfer coefficient by a, the number of electrons take part in reaction by n, current density by j, faraday constant by F. The 2.303RT/anF value refers to the slope.

- 1. **Electrochemically active surface area**

The CV was taken in an extremely narrow potential range in the non-faradaic region at different scan rates of 5-50 mVs^−1^ for the determination of electrocatalytic active surface area (ECSA). The slope value was calculated using linear fitting of the anodic and cathodic current density differential (Δj = j_anodic_ - j_cathodic_) versus the scan rate. The ECSA, which shows the existence of widely exposed catalytic active sites on the electrode material, would increase with a larger C_dl_ value. The formula used for ECSA calculation is given by the equation.

*C_dl_* = *slope*/2 eqn. S4

$ECSA=\frac{Cdl}{Csp}$ eqn. S5

whereas C_dl_ is the calculated double layer capacitance and C_sp_ is the specific capacitance taken from the literature to be equal to ~0.040 mFcm^−2^.

- - 1. **Calculation for electrochemical active area of Co_3_O_4_/SP-CN**

Straight-line equation derives from “Figure 6A & B” is shown in.

y = 0.0112x + 0.031 R² = 0.9981

*C_dl_* = *slope*/2 = 11.2/2 = 5.6 mF cm^-2^ eqn. S6

Slope = linear fit between the scan rate vs current density

Electro-active surface area = *C_dl_/C_sp_* eqn. S7

= 5.6/0.04 = 140 cm^2^

- - 1. **OH− ion adsorption capability of the developed electrodes.**

The Laviron equation is given below.

$$Ec=E\frac{1}{2}-\left( \frac{RT}{\alpha nF} \right)*\ln\left( \frac{\alpha nF}{RTks} \right)-\left( \frac{RT}{\alpha nF} \right)*\ln\left( v \right)(eqn. S10)$$

Where Ec and E1/2 are the reduction and formal potential of metal redox T, F, ks and R, stand for absolute temperature, Faraday constant, redox constant, and general gas constant, respectively. Whereas n and α represent the number of electrons transferred and electron transfer coefficient

**Table S1.** The RMSE of train, validation and test dataset along with R^2^ of each ML regression model.

| **Model** | **Train RMSE** | **Validation RMSE** | **Test RMSE** | **R^2^ Score** |
| --- | --- | --- | --- | --- |
| Linear Regression | 3.51 | 2.96 | 4.25 | 0.87 |
| Ridge Regression | 4.92 | 4.92 | 5.17 | 0.81 |
| Random Forest Regression | 0.80 | 2.65 | 3.25 | 0.92 |
| Gradient Boosting Regression | 1.06 | 2.89 | 3.36 | 0.92 |
| K-nearest neighbor Regression | 4.57 | 4.83 | 6.85 | 0.66 |
| XGBoost Regression | 0.00 | 2.34 | 2.81 | 0.94 |
| Linear Regression | 3.51 | 2.96 | 4.25 | 0.87 |
| Ridge Regression | 4.92 | 4.92 | 5.17 | 0.81 |

**
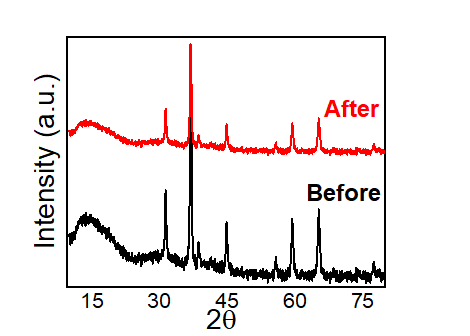
**

**Figure S4** shows comparative XRD spectrum before and after the stability test.


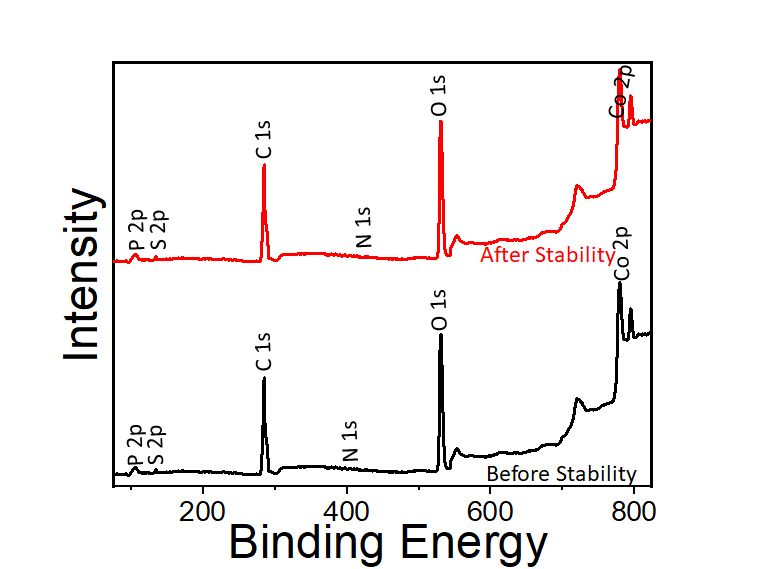


**Figure S5** shows XPS of Co₃O₄/SP-CN before and after the stability test.

**Table S2** shows a comparison table of Tafel slope and Overpotential with already reported electrodes.

| Sr. No. | Material | Tafel slope (mV dec^−1^) | Overpotential (mV) | Reference |
| --- | --- | --- | --- | --- |
| 1 | Co_3_O_4_–NiO | 55 | 280 | [1] |
| 2 | NiCo-LDH/Ar | 45 | 299 | [2] |
| 3 | Fe/Co_3_O_4_ | 45 | 350 | [3] |
| 4 | Co-Fe_2_O_3_ | 76 | 322 | [4] |
| 5 | Ni_0.7_Co_0.3_O_x_ | - | 394 | [5] |
| 6 | Co_3_Fe_7_O_x_/NPC^*^ | 61.2 | 328 | [6] |
| 7 | CoO_x_/NPC | 67.95 | 430 | [6] |
| 8 | PI/CNT^*^-Co(OH)_2_ | 49 | 317 | [7] |
| 9 | Mn_1.5_Co_1.5_ | 91 | 254 | [8] |
| 10 | Co₃O₄/SP-CN | 66 | 262 | This work |

Nitrogen-enriched porous carbon, carbon nanotube/polyimide film,

**References**

1. Srinivasa, N., et al., *Studies on Co3O4–NiO nanocomposites for potential electrocatalyst for alkaline water electrolysis.* Applied Physics A, 2022. **128**(2): p. 158.

2. Vazhayil, A., et al., *A comprehensive review on the recent developments in transition metal-based electrocatalysts for oxygen evolution reaction.* Applied surface science advances, 2021. **6**: p. 100184.

3. Iwakura, C., A. Honji, and H. Tamura, *The anodic evolution of oxygen on Co3O4 film electrodes in alkaline solutions.* Electrochimica Acta, 1981. **26**(9): p. 1319-1326.

4. Samanta, A. and S. Jana, *Ni-, Co-, and Mn-doped Fe2O3 nano-parallelepipeds for oxygen evolution.* ACS Applied Nano Materials, 2021. **4**(5): p. 5131-5140.

5. Chi, J., et al., *Nickel/cobalt oxide as a highly efficient OER electrocatalyst in an alkaline polymer electrolyte water electrolyzer.* RSC advances, 2016. **6**(93): p. 90397-90400.

6. Lin, X., et al., *Precious-metal-free Co–Fe–O x coupled nitrogen-enriched porous carbon nanosheets derived from Schiff-base porous polymers as superior electrocatalysts for the oxygen evolution reaction.* Journal of Materials Chemistry A, 2016. **4**(17): p. 6505-6512.

7. Jiang, Y., et al., *Enhanced electrocatalytic oxygen evolution of α-Co (OH) 2 nanosheets on carbon nanotube/polyimide films.* Nanoscale, 2016. **8**(18): p. 9667-9675.

8. Chinnadurai, D., et al., *Mn-Co bimetallic phosphate on electrodeposited PANI nanowires with composition modulated structural morphology for efficient electrocatalytic water splitting.* Applied Catalysis B: Environmental, 2021. **292**: p. 120202.
